# From arginine methylation to ADMA: A novel mechanism with therapeutic potential in chronic lung diseases

**DOI:** 10.1186/1471-2466-9-5

**Published:** 2009-01-29

**Authors:** Dariusz Zakrzewicz, Oliver Eickelberg

**Affiliations:** 1 University of Giessen Lung Center, Department of Medicine II, Aulweg 123, 35392 Giessen, Germany; 2 Comprehensive Pneumology Center, Munich, Germany; 3 Institute of Lung Biology and Disease, Helmholtz Zentrum München, Munich/Neuherberg, Germany

## Abstract

Protein arginine methylation is a novel posttranslational modification regulating a diversity of cellular processes, including protein-protein interaction, signal transduction, or histone function. It has recently been shown to be dysregulated in chronic renal, vascular, and pulmonary diseases, and metabolic products originating from protein arginine methylation have been suggested to serve as biomarkers in cardiovascular and pulmonary diseases.

Protein arginine methylation is performed by a class of enzymes called protein arginine methyltransferases (PRMT), which specifically methylate protein-incorporated arginine residues to generate protein-incorporated monomethylarginine (MMA), symmetric dimethylarginine (SDMA), or asymmetric dimethylarginine (ADMA). Upon proteolytic cleavage of arginine-methylated proteins, free intracellular MMA, SDMA, or ADMA is generated, which, upon secretion into the extracellular space (including plasma), directly affects the methylarginine concentration in the plasma. Free methylarginines are cleared from the body by renal excretion or hepatic metabolism. In addition, MMA and ADMA, but not SDMA, can be degraded via a class of intracellular enzymes called dimethylarginine dimethylaminohydrolases (DDAH).

ADMA and MMA are endogenous inhibitors of nitric oxide synthases (NOS) and ADMA has been suggested to serve as a biomarker of endothelial dysfunction in cardiovascular diseases. This view has now been extended to the idea that, in addition to serum ADMA, the amount of free, as well as protein-incorporated, intracellular ADMA influences pulmonary cell function and determines the development of chronic lung diseases, including pulmonary arterial hypertension (PAH) or pulmonary fibrosis. This review will present and discuss the recent findings of dysregulated arginine methylation in chronic lung disease. We will highlight novel directions for future investigations evaluating the functional contribution of arginine methylation in lung homeostasis and disease with the outlook that modifying PRMT or DDAH activity presents a novel therapeutic option for the treatment of chronic lung disease.

## A brief introduction to protein arginine methylation

During the last 40 years, arginine methylation has been extensively studied in prokaryotes and eukaryotes, revealing a pivotal role of this posttranslational modification in the regulation of a number of cellular processes. Protein arginine methylation is involved in the modulation of transcription, RNA metabolism, or protein-protein interaction, thereby controlling cellular differentiation, proliferation, survival, or apoptosis [1,2].

**Figure 1:**
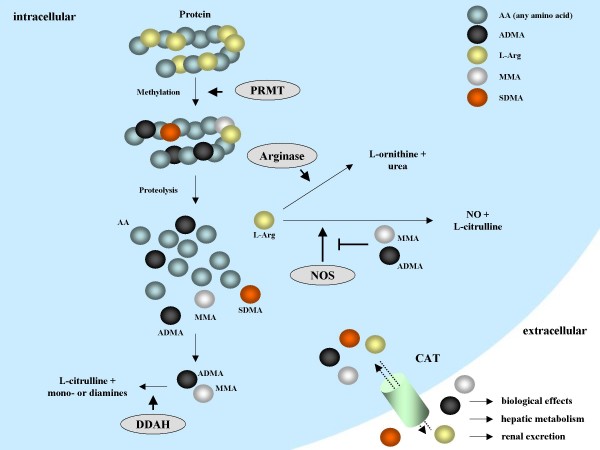
**Methylarginine metabolism**. Protein arginine methylation is performed by a class of enzymes termed protein arginine methyltransferases (PRMT), which specifically methylate protein-incorporated L-arginine (L-Arg) residues to generate protein-incorporated monomethylarginine (L-MMA), asymmetric dimethylarginine (ADMA), or symmetric dimethylarginine (SDMA). Upon proteolytic cleavage of arginine-methylated proteins, free intracellular MMA, ADMA, or SDMA are generated. Free L-Arg can be metabolized by arginases to L-ornithine and urea, or by nitric oxide synthases (NOS) to NO and L-citrulline. Free methylarginines can also be released to the extracellular space by cationic amino acid transporters (CAT) to induce distinct biological effects, undergo hepatic metabolism, or renal excretion. MMA and ADMA, but not SDMA can be converted to L-citrulline and mono- or diamines by a class of intracellular enzymes called dimethylarginine dimethylaminohydrolases (DDAH). Most importantly, MMA and ADMA, but not SDMA, act as potent endogenous inhibitors of NOS enzymes.

The methylation of protein arginine residues is catalyzed by a family of intracellular enzymes termed protein arginine methyltransferases (PRMT) [2] (Figure 1). In mammalian cells, these enzymes have been classified into type I (PRMT1, 3, 4, 6, and 8) and type II PRMT (PRMT5, 7, and FBXO11), depending on their specific catalytic activity. In addition, PRMT2 was identified as a methyltransferase most probably belonging to type I enzymes, but its methyltransferase activity has yet not been unequivocally characterized [2]. Both types of PRMT, however, catalyze the formation of mono-methylarginine (MMA) from L-arginine (L-Arg). In a second step, type I PRMT produce asymmetric dimethylarginine (ADMA), while type II PRMT form symmetric dimethylarginine (SDMA) [1,2]. After proteolytic degradation of methylated intracellular proteins, free MMA, SDMA, or ADMA can be released from cells (Figure 1). Thus, protein degradation represents the major source of free intracellular methylarginines, as there is currently no evidence that free L-Arg can be methylated [3,4]. In addition, intracellular proteolysis of methylated proteins also significantly contributes to interstitial and plasma ADMA levels, which are further controlled by degradation and cellular export/import of methylarginines. Released ADMA can also be taken up by other cells via the cationic amino acid (y^+^) transporters, which are widely expressed in mammalian cells [5](Figure 1).

Free methylarginines are cleared from the body by renal excretion and hepatic metabolism [3,4]. In addition, MMA and ADMA, but not SDMA, can be degraded to citrulline and mono- or dimethylamines, respectively, by dimethylarginine dimethylaminohydrolases (DDAH) [3]. To date, two DDAH isoforms have been cloned and characterized, termed DDAH1 and DDAH2 [3]. Alternatively, ADMA can also be converted to α-keto valeric acid by alanine:glyoxylate aminotransferase 2 [6], although the influence of this pathway on total ADMA metabolism has not been extensively studied thus far. Of interest, the demethylation/clearance of methylarginines is restricted to free methylarginines, as a theoretically possible demethylation of protein-incorporated, methylated L-Arg residues *in situ *has yet not been demonstrated. It should be noted, however, that the conversion of protein-incorporated MMA to citrulline by peptidylarginine deiminase 4 was recently demonstrated, which prevented histone methylation by PRMT 1 and 4 [7,8]. This may influence protein methylation directly, as MMA deimination will decrease the amount of protein-incorporated MMA that is available for dimethylation by PRMT, but the relevance of protein deimination of protein-incorporated MMA by PAD enzymes has also been challenged lately [9,10]. Finally, free SDMA has been described to be catabolized *in vivo *when injected intraperitoneally into rats, although the enzymes involved have thus far not been identified [11].

ADMA has been detected in urine, plasma, cerebrospinal and bronchoalveolar lavage (BAL) fluids, and various types of tissues [3,4,12,13]. Specific methylation of protein-incorporated L-Arg residues was originally described in 1968 [14], but the key finding that ADMA is a potent inhibitor of all three nitric oxide synthase (NOS) isoforms (nNOS, iNOS, and eNOS), resulting in impaired NO production *in vitro *and *in vivo*, was only reported in 1992 by Vallance et al. [15]. NO is a well-known vasodilator that essentially controls a diverse range of pulmonary functions, such as macrophage activity, pulmonary artery vasodilation, or bronchoconstriction [16]. ADMA may therefore control pulmonary cell functions either via direct effects on gene expression and protein function, as recently shown in an elegant study [17], or via inhibition of NOS and subsequently altered NO generation. Furthermore, the lung generates a significant amount of ADMA itself, and as such may directly contribute to interstitial and plasma ADMA levels [18], further suggesting that dysregulated ADMA metabolism in the lung may trigger, initiate, or perpetuate chronic lung diseases, such as pulmonary arterial hypertension (PAH), idiopathic pulmonary fibrosis (IPF), asthma, or chronic obstructive pulmonary disease (COPD). In the following, we will outline the current evidence of dysregulated protein arginine methylation or ADMA levels in specific lung diseases.

## Arginine methylation in pulmonary arterial hypertension

Pulmonary arterial hypertension (PAH) is a fatal syndrome characterized by an elevated blood pressure in the pulmonary circulation, due to increased resistance of pulmonary arterioles [19]. The pathophysiology of PAH includes endothelial dysfunction and pulmonary arterial smooth muscle cell (PASMC) hypertrophy and proliferation [20]. Elevated ADMA concentrations have been detected in the plasma of patients with idiopathic (I)PAH [21-23], chronic thromboembolic pulmonary hypertension (CTEPH) [24], or PAH related to sickle cell disease [25] or systemic sclerosis [26], suggesting a strong association of circulating methylarginine levels with PAH pathogenesis. A recent study by Xu et al., however, detected increased arginase activity in the serum of PAH patients, whereas serum ADMA levels did not differ between PAH patients and healthy individuals, suggesting that increased arginase activity was responsible for endothelial dysfunction observed in PAH [27]. This is of particular interest, as arginase directly metabolizes free L-Arg. Increased arginase activity as well as ADMA content was subsequently confirmed in pulmonary arterial endothelial cells in the rat model of monocrotaline-induced pulmonary hypertension [28].

The investigation of animal models of PAH, using e.g. chronic hypoxia or the pyrrolizidine alkaloid monocrotaline to induce PAH in piglets, mice, or rats, have largely detected increased plasma ADMA during the development of experimental PAH, but have thus far suggested different mechanisms responsible for this increase. While some groups have reported decreased DDAH1 expression along with increased ADMA levels [28,29], others have detected decreased DDAH2 in this condition [22,30]. Most importantly, the analysis of lung tissues obtained from IPAH or control patients have demonstrated an impaired expression of DDAH2, but not DDAH1, in IPAH [22]. Furthermore, PRMT expression was found to be upregulated in mice exposed to chronic hypoxia, resulting in increased ADMA tissue levels and a decreased L-Arg/ADMA ratio, thereby supporting an important role of PRMT-mediated ADMA generation in hypoxia-induced PAH [31].

In sum, disrupted methylarginine metabolism most likely impairs vascular homeostasis in PAH, but it remains unclear, which DDAH or PRMT isoforms control ADMA tissue and plasma levels under pathological conditions. Further, the relative contributions of serum, interstitial, or pulmonary ADMA levels to PAH pathogenesis remain unclear, as comparative investigations are still lacking. Data obtained in transgenic mice with manipulation of DDAH expression have thus far supported a major role for DDAH1 in serum and tissue ADMA homeostasis. Forced overexpression of DDAH-1 in transgenic mice resulted a 2-fold reduction in plasma ADMA levels and decreased blood pressure [32], while the loss of DDAH-1 activity by homologous recombination in mice led to accumulation of circulating ADMA and increased blood pressure [33]. In contrast, forced overexpression of DDAH2 led only to a 20% reduction in circulating ADMA, but no reduction in systemic blood pressure [34].

## Arginine methylation in pulmonary fibrosis

Compared with the aforementioned available data on ADMA and vascular remodeling in PAH, much less is known about the role of methylated arginines in interstitial remodeling of the lung, e. g. in idiopathic pulmonary fibrosis (IPF). A growing body of evidence, however, suggests that arginine methylation and ADMA metabolism may be involved in the progression of IPF, a lethal disorder of major concern due to its unresolved pathogenesis and limited responsiveness to currently available therapies [35]. The hallmark lesions of IPF are fibroblast foci, which are sites featuring α-smooth muscle actin (αSMA)-positive, activated (myo)fibroblasts that synthesize and deposit a collagen-rich extracellular matrix [36]. Fibroblast foci occur in subepithelial layers adjacent to areas of alveolar epithelial cell injury, suggesting that altered epithelial-mesenchymal crosstalk contributes to the pathobiology of IPF. Indeed, it is well accepted that repetitive alveolar epithelial cell injury and subsequent repair, in the presence or absence of local inflammation, represents a key pathogenic mechanism in IPF [35,36]. This leads to aberrant growth factor activation and perpetuation of fibrotic transformation. In addition to the well-described cytokines and growth factors interleukin-4, -13, -21, wingless, or transforming growth factor-β, components of the renin-angiotensin-aldosterone system including angiotensin II (ANGII) have recently been identified as important regulators of fibrosis [37,38].

**Figure 2:**
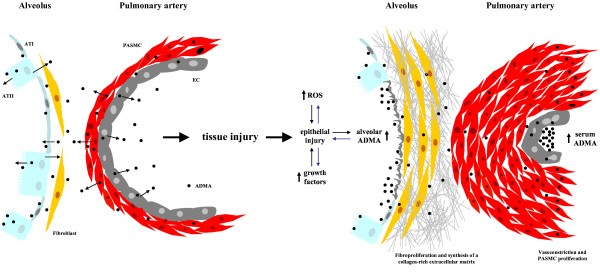
**Abnormal protein arginine methylation triggers pathological changes in the lung**. In the normal lung, methylarginines are generated via intracellular proteolysis and released to the intraalveolar, interstitial, and intravascular compartments of the lung. Pathological tissue injury, in particular alveolar epithelial cell injuries, leads to an increase of reactive oxygen species (ROS), growth factor production. This perpetuates epithelial cell damage and leads to increases of alveolar ADMA via increased proteolysis. Secondary pathological events of lung tissue injury include, but are not restricted to, fibroproliferation and deposition of extracellular matrix, as well as vasoconstriction and pulmonary artery smooth muscle cell (PASMC) proliferation. ATI; type I alveolar epithelial cell, ATII; type II alveolar epithelial cell type II, EC; endothelial cell.

Interestingly, ANGII infusions increased plasma ADMA levels and caused perivascular and interstitial renal fibrosis [34,39]. Overexpression of DDAH1/2 protected from ANG II-induced ADMA increases and interstitial fibrosis [34,39]. These data suggest an delicate causal relationship between ANGII-ADMA and the development of perivascular and interstitial fibrosis, which has to be further explored in human IPF in future studies. Most importantly, direct infusion of ADMA resulted in elevated collagen deposition in mouse lungs and enhanced arginase activity [40], a feature of experimental lung fibrosis [41]. In this context, it is important to note that preterm infants requiring mechanical ventilation exhibited higher ADMA plasma levels than preterm infants who did not require mechanical ventilation [42]. As such, lung damage as evident in preterm infants under mechanical ventilation may lead to increased ADMA release via enhanced proteolysis and cell death. It is therefore reasonable to state that elevated serum/alveolar/pulmonary ADMA levels lead to vascular and/or interstitial remodeling in the lung, in the presence or absence of inflammation (Figure 2), but the causal relationship between lung injury, ADMA metabolism, and remodelling remains to be dissected in detail in future studies.

## Arginine methylation in COPD

It has recently been suggested that NO metabolites, reactive oxygen species, and nitrosothiols represent novel targets for the prevention and treatment of chronic inflammatory airway diseases including asthma and chronic obstructive pulmonary disease (COPD) [43,44]. In contrast, little is known about methylarginine metabolism in the pathogenesis of these disorders. Since cigarette smoke represents the main risk factor for COPD, several studies have investigated the relationship between cigarette smoke and ADMA levels, with conflicting results. While some studies have found decreased ADMA levels in smokers compared with non-smokers [45,46], others have detected increased ADMA levels in smokers, in the absence or presence of triple vessel coronary artery disease [47,48].

A similar divergence is evident in *in vitro *studies using cigarette smoke extract (CSE)-treated cell cultures. The exposure of human endothelial cells to 10% CSE decreased intracellular ADMA concentration via increased DDAH2 expression [46], while others have reported increased ADMA levels under such conditions [48]. In the absence of direct ADMA measurements in COPD or asthma patients, the available data therefore suggests that cigarette smoke is an important regulator of ADMA synthesis, but more conclusive *in vivo *and *in vitro *evidence is needed to assess whether COPD or asthma, or pathogenetic aspects thereof, are associated with altered ADMA levels.

## The relevance of plasma ADMA levels

A significant debate about the contribution of plasma ADMA to the regulation of NOS-dependent NO production has recently been initiated. In pathological conditions including PH, plasma ADMA levels have been shown to increase to 0.53 ± 0.15 μM (from 0.36 ± 0.05 μM in controls) and 1.06 ± 0.06 μM (from 0.48 ± 0.04 μM) in studies by Kielstein et al. and Pullamsetti et al. [22,23], respectively. Taking into consideration the physiological plasma levels of L-Arg of 100 μM, the normal plasma ADMA concentrations of 0.42 ± 0.06 μM [18] must approach approximately 10 μM to elicit a significant effect on NO production and physiological functions thereof in the plasma [49]. It is therefore highly unlikely that plasma ADMA levels significantly contribute to decreased serum NO bioavailability observed in PAH or other cardiovascular disorders. In contrast, intracellular ADMA levels may significantly influence NOS activity under pathological conditions, as these have been shown to increase from 5.8 ± 1.2 μM to 21.6 ± 4.7 μM, for example, in carotid arteries subjected to balloon-induced vascular injury [49]. Increased intracellular ADMA levels may be particularly relevant to lung diseases, as the lung exhibits one of the highest baseline concentrations of intracellular ADMA [18]. Of note, small changes in plasma ADMA may serve as indicators of greater changes in intracellular ADMA. Therefore, further studies should closely investigate alterations of the intracellular methylarginine content in chronic lung disease, a factor that is clearly more likely to modify NO generation. In contrast, altered plasma ADMA levels may rather be a marker of disrupted methylarginine metabolism in selected intracellular compartments rather than a direct cause of structural or functional abnormalities in the cardiovascular system.

## Outlook

In conclusion, dysregulated arginine methylation has now been shown to contribute to the pathogenesis of several pulmonary disorders, in experimental animal models as well as human disease. Causal relationships between dysregulated arginine methylation and the initiation, progression, or therapy of lung disease, however, remain to be dissected. Future investigations of arginine methylation dynamics in these diseases will therefore have to address, among others, the following questions:

1) Which tissues are the source of increased plasma ADMA levels in chronic lung disease?

2) Which specific cell types are the major contributors within a given tissue to altered plasma ADMA levels under pathophysiological conditions?

3) Can dysregulated arginine methylation itself lead to altered lung structure and function?

Future studies will undoubtedly shed light on the relative importance of protein arginine methylation and highlight cellular events that are controlled by posttranslational modification of L-Arg residues under physiological and pathophysiological scenarios. The regulation of methylarginine metabolism by modulating cellular PRMT or DDAH activity will therefore likely present a novel therapeutic option for the treatment of chronic lung diseases such as PAH, IPF, asthma, or COPD.

## Statement of competing interests

The authors declare that they have no competing interests.

## Pre-publication history

The pre-publication history for this paper can be accessed here:


